# Diabetes Risk Assessment Among the City Population in Azad Kashmir: A Cross-sectional Study

**DOI:** 10.7759/cureus.4580

**Published:** 2019-05-01

**Authors:** Arslaan Javaeed, Ujyara Maryam Lone, Saima Sadiq, Sanniya Khan Ghauri, Zarghoona Wajid

**Affiliations:** 1 Pathology, Poonch Medical College, Rawalakot, PAK; 2 Emergency Medicine, Shifa International Hospital, Islamabad, PAK; 3 Internal Medicine, Dr. Ziauddin Hospital, Karachi, PAK

**Keywords:** type 2 diabetes mellitus, risk, ausdrisk tool, azad kashmir

## Abstract

Objective

To determine the frequency of people at risk of developing diabetes mellitus type 2 (DMT2) and their risk of developing the disease over the next five years, using the Australian type 2 diabetes risk assessment (AUSDRISK) tool.

Methods

A cross-sectional study was done involving 152 adults; both males and females were randomly selected from city populations in Rawalakot and Muzaffarabad of the Azad Kashmir, irrespective of weight, family history and dietary habits. Patients with the apparent clinical features of DMT2 were excluded from the study. Data were collected over a nine-month period from April 2017 using an interviewer-administered questionnaire based on the AUSDRISK tool.

Results

Statistical analysis was done using SPSS version 23.0 (IBM, Armonk, NY, USA). Descriptive statistics were used to calculate the frequencies and percentages. Fifty-four (35.5%) participants had a low risk, 88 (57.9%) had an intermediate risk, and 10 (6.6%) had a high risk of developing DMT2 over the next five years.

Conclusion

Most of the city occupants had an intermediate-to-high risk of developing DMT2 (64.5%) over the next five years.

## Introduction

Diabetes mellitus (DM) is a leading cause of morbidity in the world, with Pakistan having a high burden of disease. The trend is bound to become worse with time [1]. However, this can be slowed down by instituting preventive measures in people with a high risk of developing diabetes mellitus type II (DMT2) which can be assessed using a highly sensitive and specific DMT2 risk assessment tool like the Australian type 2 diabetes risk assessment (AUSDRISK) tool [2,3].

DM is a common chronic metabolic disease that is characterized by hyperglycaemia arising from absolute or relative insulin deficits in production or its actions [4]. The World Health Organization (WHO) estimates that approximately 422 million people suffer from the disease worldwide, with the numbers expected to double by the year 2030 [5, 6]. Differentiating the two types of DM (type I and type II) is challenging and so are the statistics differentiating the two entities [7]. DMT2 affects more people worldwide than diabetes mellitus type I (DMT1). People in Central and South Asia are at an increased risk of developing diabetes compared to other races due to their genetic make-up, early life environmental influences and family history of diabetes [8]. The estimated prevalence of DMT1 in urban Pakistan is 6.0% among males and 3.5% among females [9]. A literature review done by Meo et al. places the average prevalence of type 2 diabetes at 11.77% in Pakistan [10].

This high burden of disease has led to the development of screening protocols to identify people at risk of DMT2 and thus provide timely interventions to halt progression of the disease by enrolling them into preventive programmes, or start early treatment of the disease and delay the onset of complications. Screening involves testing asymptomatic individuals with higher risk of the diseases. A desirable screening tool must be minimally invasive and have a high sensitivity rate to identify most of those people at risk. Given that Pakistan is a developing country where few people are likely to volunteer for diagnostic testing in early stages of the disease, screening provides for a cost-effective way of reducing the burden of disease [11].

Since July 2008, one such tool used in the assessment of the risk of developing diabetes is the AUSDRISK. AUSDRISK scores were calculated from patients aged 40-74 years in South West Victoria, Australia. The study showed that increased scores were indicative of an increase in the patients’ weight, body mass index (BMI), fasting blood sugar and risk of metabolic syndrome. A score of 12 or more indicated that the patient had a high risk of developing diabetes type 2 over a 10-year period. This was identified in 39.5% of the study population [2]. A similar study was designed to identify the characteristics of men categorized as having a high risk of developing diabetes using the AUSDRISK assessment tool. The researchers concluded that the men were older than 44 years of age, had a large waist circumference, and elevated hemoglobin A1c (HBA1c) indicating a high risk for diabetes type 2 [3].

The AUSDRISK tool had a higher sensitivity (80.3%, 95% confidence interval of 76.6%-84.5%) and specificity (78.1%, 95% confidence interval 76.9-79.2%) compared to other pre-existing tools such as the Finnish Diabetes Risk Score (FINDRISC) assessment tool with a sensitivity of 67.7%, specificity of 67.2% and a 51% positive predictive value [12]. Thus, the AUSDRISK assessment tool is an effective tool that can be used to estimate the risk of developing DMT2.

## Materials and methods

This cross-sectional epidemiologic study was conducted in Rawalakot and Muzaffarabad from April 2017 to January 2018. The population of Azad Kashmir is 4,045,366 according to the 2017 census. The study population was comprised of adults aged from 18 to 60 years from all occupations. The sample was calculated using the WHO sample size calculator as shown [13]. The formula used in the calculation was n = Z2(p * q)/d2, where, n = desired sample size, Z = standard deviation (1.96 at 95% confidence interval), p = prevalence, q = 1 - p and d = errors allowed in the study (5% = 0.05).

**Figure 1:**
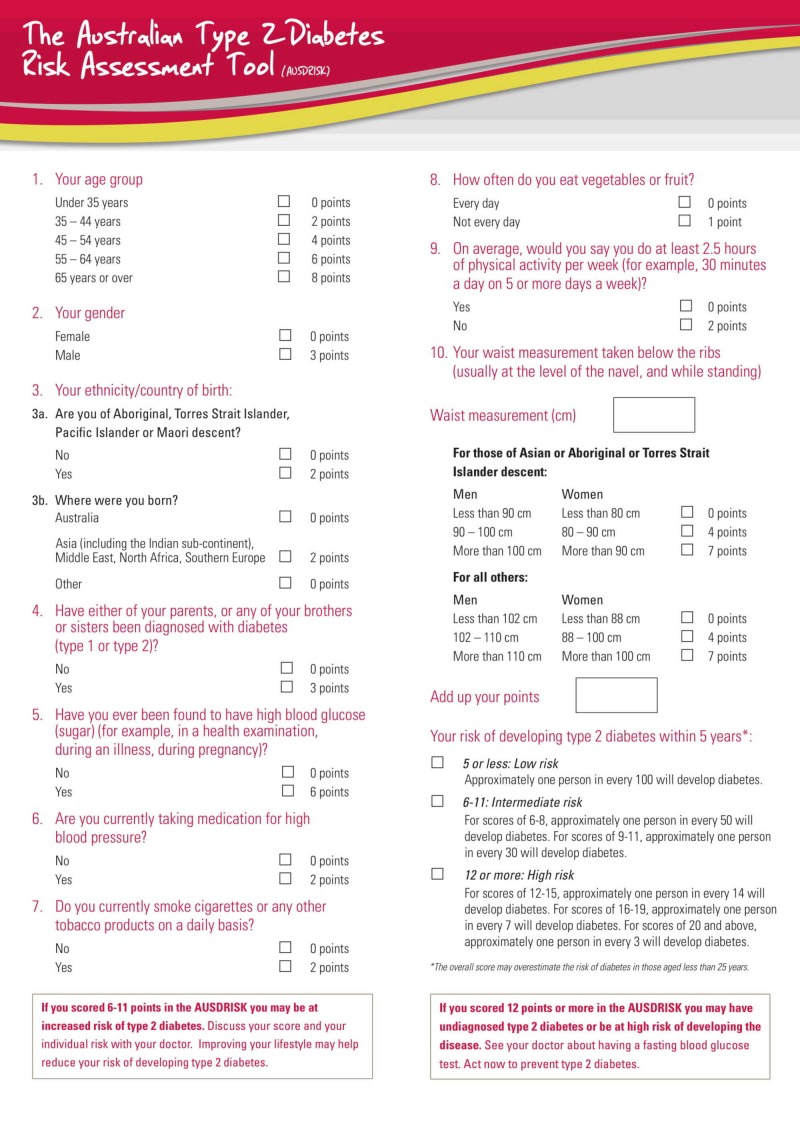
The Australian Type 2 Diabetes Risk Assessment Tool

One hundred and fifty-two participants were recruited in the study using the non-probability consecutive sampling technique. Ethical approval was sought from the Ethical Review Board of Poonch Medical College, Rawalakot (PMC/RKT/EA/04/17). The objectives of the research were communicated to each participant and informed written consent was secured. Adults (age 18 to 60 years) without diagnostic symptoms of DMT2 (polyuria, polydipsia and polyphagia) were recruited from the hospitals’ outpatient clinics. The data were collected by doctors in the outpatient clinics using an interviewer-administered questionnaire based on the AUDRISK tool. The tool [14] contained 10 sets of questions that featured variables that are known to influence the occurrence of diabetes type 2. They include age, gender, ethnicity, family history of diabetes, smoking, dietary intake, hypertension, obesity and prediabetic states as shown in Figure 1.

The questions were answered appropriately, and corresponding scores attached to each question. This was followed by the calculation of a summated score and analysis to determine the association between the risk factors under study and the five-year risk of developing diabetes.

Categorical variables were presented as frequencies and percentages. The relationship between gender and risk factors of DMT2 was calculated using the Chi-squared test. The analysis was performed with a 95% confidence interval using Statistical Package for Social Science (SPSS), version 23.0 (IBM, Armonk, NY, USA).

## Results

Out of 152 study participants, 60 (39.5%) were males and 92 (60.5%) were females. The study showed that 54 (35.5%) of participants had low risk, 88 (57.9%) had intermediate risk, and 10 (6.6%) had high risk of developing DMT2. There were more males (07) than females (03) with a high risk of developing DMT2, despite the majority of the respondents being female, thus indicating that male respondents had a relatively higher risk of developing DMT2 compared to their female counterparts. The most important observation is in all sexes, the highest percentage of respondents had an intermediate risk, i.e., 28 (46.6%) males and 60 (65.2%) females. A third of males and females had a low risk of developing DMT2 as shown in Table 1.

**Table 1:** Male and female population at low, intermediate, and high level of developing type 2 diabetes mellitus

5 or less: Low risk				6-11: Intermediate risk				12 or more: High risk			
Males		Females		Males		Females		Males		Females	
N	%	N	%	N	%	N	%	N	%	N	%
25	41.7	29	31.5	28	46.6	60	65.2	07	11.7	03	3.3

Analysis of the relationship between gender and risk factors for DM showed that males had a higher mean weight at 68 ± 15.51 kg compared to 55 ± 12.66 kg in females. Similarly, males had a higher mean height at 165 ± 8.35 cm compared to 150 ± 7.55 cm among females and a higher waist circumference at 86 ± 14.65 cm compared to 78 ± 12.25 cm recorded by the female respondents. Lastly, males had a higher mean recorded for their BMI at 26 ± 6.25 kg/m2 compared to 22 ± 5.35 kg/m2 among females. The summary of the risk factors for DMT2 by age is shown in Table 2 below, thus indicating that most of the males were overweight and had a higher risk of developing DMT2 compared to females.

**Table 2:** Relationship between gender and risk factors for diabetes mellitus BMI: Body Mass Index

Risk factors	Males Mean ± SD	Females Mean ± SD	p-value
Weight in kg	68 ± 15.51	55 ± 12.66	0.021
Height in cm	165 ± 8.35	150 ± 7.55	0.001
BMI in kg/m^2^	26 ± 6.25	22 ± 5.35	0.001
Waist circumference in cm	86 ± 14.65	78 ± 12.25	0.004

## Discussion

This study found out that there was an intermediate-to-high risk of developing diabetes mellitus type II among the participants. The study compares well with other studies done in Pakistan in that most studies entail patients aged 18-60 years. However, one previous study where risk assessment was done using a different tool (FINDRISC tool), females comprised a minority (43.2%) of the respondents as compared to this study that had 60.5% of the participants being female [15, 16].

On the summated risk of developing DMT2, the study showed that 54 (35.5%) of participants had a low risk, 88 (57.9%) had an intermediate risk, 10 (6.6%) had a high risk of developing DMT2; this contrasts with the 39.5% of respondents with a high risk in a study done in South West Victoria using a similar tool [16]. The difference in populations in Australia having a higher prevalence of the disease and a higher prevalence of risk factors is due to stringent screening protocols in Australia as compared to Pakistan. In the FINDRISC study carried out among Pakistani squatters, there were equal proportions of respondents classified as having a high risk of developing DMT2, i.e., 6.6% in this study compared to 7.01% who had a high risk and a very high risk of developing DMT2 in the FINDRISC study. In all studies, the majority of the respondents had a low and intermediate risk of developing DMT2 [2,17].

Anthropometric analysis indicated that the city population was identical to the squatter population in terms of mean weight and height of both sexes. In both studies, males had a higher mean height (170.2 ± 6.31 cm and 165 ± 8.35 cm) compared to females (156.8 ± 7.09 cm and 150 ± 7.55 cm) and a higher mean weight (71.2 ± 13.5 and 68 ± 15.51 kg) compared to females (67.4 ± 15.61 kg and 55 ± 12.66 kg). This concurs with the fact that Pakistani males are at a higher risk of developing DMT2 in both studies and such conclusions were justified from the data obtained in these studies. However, men from the city population had a higher BMI than females which was different from the squatter population. This could be attributed to the lifestyle of city populations where both sexes are engaged in physical activities at their workplaces and recreational physical activities are pursued by city women compared to women of the squatter population who were largely home managers [3, 17].

## Conclusions

The current study showed higher percentage of males with high risk of diabetes mellitus compared to females (11.7% vs 3.3%) whereas intermediate risk of diabetes was observed in more females (65.2%) than males (46.6%). Overall, more than half of the population had an intermediate-to-high risk of developing DMT2 over a five-year period. Further large-scale randomized controlled trial (RCT) may predict the risks more precisely.
